# Carotid Intima-media Thickness and its Association with Conventional Risk Factors in Low-income Adults: A Population-based Cross-Sectional Study in China

**DOI:** 10.1038/srep41500

**Published:** 2017-01-30

**Authors:** Bin Liu, Jingxian Ni, Min Shi, Lingling Bai, Changqing Zhan, Hongyan Lu, Yanan Wu, Jun Tu, Xianjia Ning, Junwei Hao, Jinghua Wang

**Affiliations:** 1 Department of Ultrasound, Tianjin Huanhu Hospital, Tianjin, China, 300350, China; 2 Department of Neurology, Tianjin Medical University General Hospital, Tianjin, 300052, China; 3 Department of Epidemiology, Tianjin Neurological Institute, Tianjin 300052, China; 4 Department of Neurology, Wuhu Second People’s Hospital, Wuhu, Anhui 241000, China; 5 Center of Epidemiology, Tianjin Medical University General Hospital, Tianjin, 300052, China

## Abstract

Carotid intima-media thickness (CIMT) is an established predictor of cardiovascular disease and stroke. However, risk factors associated with CIMT remain unclear. Therefore, we aimed to identify factors associated with CIMT in a low-income Chinese population. Stroke-free and cardiovascular disease-free residents aged ≥45 years were recruited. B-mode ultrasonography was performed to measure CIMT. The mean age of participants (n = 3789) was 59.92 years overall, 61.13 years in men, and 59.07 years in women (P < 0.001). Male sex, older age, low education level, smoking, hypertension, and high systolic blood pressure, fasting blood glucose, and low-density lipoprotein cholesterol levels were independent determinants of mean CIMT. Mean CIMT was higher by 18.07 × 10^−3^ mm in hypertensive compared to normotensive participants (P < 0.001), by 19.03 × 10^−3^ mm in men compared to women (P < 0.001), and by 9.82 × 10^−3^ mm in smokers compared to never smokers (P < 0.001). However, mean CIMT decreased by 1.07, 0.37, and 2.36 × 10^−3^ mm per 1-unit increase in education level, diastolic blood pressure, and triglycerides, respectively. It is important to manage conventional risk factors in low-income populations to decrease stroke incidence.

Globally, the age-standardised incidence rates of stroke in low- and middle-income countries exceed those of high-income countries by 23%^1^. The number of patients younger than 75 years with incident stroke in low- and middle-income countries is three times higher than that in high-income countries^1^. An estimated 6.6 million Americans ≥20 years of age were diagnosed with stroke from 2009 to 2012^2^. Furthermore, the significant reduction in the mortality rate of stroke in low-, middle-, and high-income countries has increased the burden of stroke^1^.

Statistics from a 2015 cardiovascular disease report in China revealed that cardiovascular disease (CVD), including coronary heart disease and stroke, is the leading cause of death in the Chinese population, accounting for 40% of all deaths. Of these, the proportion of CVD-related deaths accounted for 44.6% in rural areas and 42.5% in urban areas^3^. In addition, our previous studies demonstrated the greatest age-standardised incidence of first-ever stroke (318.2/100,000 person-years between 2006 and 2012) in a low-income population in rural China, along with a dramatic increase in stroke incidence over the past two decades^4^,^5^,^6^. The prevalence of conventional risk factors was high in this population and significantly increased over the past several decades^7^,^8^.

Carotid atherosclerosis has been shown to be a strong predictor of CVD and stroke^9^,^10^,^11^,^12^. Carotid intima-media thickness (CIMT), which is a useful measure for assessing CVD and stroke risks, is a non-invasive, inexpensive, rapid, and reproducible modality^13^,^14^,^15^. However, the relationship between traditional risk factors and CIMT in a low-income population with a high incidence of stroke is currently unclear.

Therefore, we aimed to evaluate the characteristics of mean CIMT according to different demographic characteristics and relevant factors associated with CIMT in a low-income population in China that had a high incidence of stroke.

## Results

### Characteristics of the participants

The selection process for participants was described in a previous publication^16^. In brief, a total of 4012 individuals were interviewed among 5380 qualified residents during the study period. The frequency of responding accounted for 75%. Finally, 3789 subjects were assessed in this study after excluding 223 residents with a previous history of CVD or stroke.

The mean age was 59.92 years overall, 61.13 years in men, and 59.07 years in women (P < 0.001). Men were more likely to have high education levels, current smoking status, consume alcohol, and have hypertension; however, women had higher prevalence rates for illiteracy and obesity. Moreover, SBP and DBP levels were higher in men than in women, but TC, TG, HDL-C, and LDL-C levels were higher in women than in men (Table 1).

### Mean CIMT according to demographics and conventional risk factors

Table 2 shows that the mean CIMT measurements were 0.57mm overall, 0.58 mm in men, and 0.56 mm in women. In the univariate analysis, the mean CIMT was associated with sex, age, education level, smoking, alcohol consumption, hypertension, diabetes, high-TG, and high-LDL-C. Of these, a lower mean CIMT was found in those with higher education levels and high-TG (all P < 0.05).

### Multiple linear regression analysis of conventional risk factors associated with mean CIMT

A multivariate analysis was performed to assess the relationship between mean CIMT and conventional risk factors. Male sex, older age, current smoking, hypertension, and high levels of SBP, FBG, and LDL-C were independent determinants of mean CIMT. Mean CIMT increased by 1.66 × 10^−3^ mm per 1-year age increase (P < 0.001), by 0.40 × 10^−3^ mm per 1-mmHg SBP increase (P < 0.001), by 2.24 × 10^−3^ mm per 1-mmol/L FBG increase (P = 0.001), and by 4.95 × 10^−3^ mm per 1-mmHg/L LDL-C increase (P = 0.003). Moreover, mean CIMT was higher by 19.03 × 10^−3^ mm in men compared to women (P < 0.001), by 9.82 × 10^−3^ mm in current smokers compared to never smokers (P = 0.027), and by 18.07 × 10^−3^ mm in hypertensive participants compared to normotensive participants (P < 0.001). However, a negative correlation was observed between mean CIMT and education level, DBP, and TG; mean CIMT decreased by1.07 × 10^−3^ mm per 1-year education level increase (P = 0.017), by 0.37 × 10^−3^ mm per 1-mmHg DBP increase (P = 0.032), and by 2.36 × 10^−3^ mm per 1-mmol/L TG increase (P = 0.035). Age had the greatest contribution to mean CIMT, with a standardized regression coefficient of 0.182 (Table 3).

## Discussion

This is the first report on the characteristics and factors associated with CIMT in a low-income population with a high incidence of stroke. Compared with previous reports from non-Asian populations, we found a lower mean CIMT in this population with a high incidence of stroke and high prevalence of conventional risk factors^4^,^5^,^6^,^7^,^8^. Positive correlations were observed between mean CIMT and older age, male sex, current smoking, SBP, FBG, and LDL-C, an inverse relationship was found between mean CIMT and education level. However, inconsistent with that seen in previous studies, a negative association between mean CIMT and DBP and TG was observed in this study.

The Gutenberg-Heart Study, which was a community-based study, reported median CIMT values of 0.62mm in women and 0.65 mm in men^17^. A significant trend of higher CIMT with increasing age was observed in a community-based study in Taiwan^18^; the mean CIMT values were 0.58 mm (35–44 years), 0.66 mm (45–54 years), 0.75 mm (55–64 years), 0.89 mm (65–74 years), and 1.00 mm (≥75 years) in men; and corresponding values were 0.52 mm, 0.61 mm, 0.68 mm, 0.79 mm, and 0.98 mm in women. The Carotid Atherosclerosis Progression Study showed quartile mean CIMT values of 0.63 mm, 0.70 mm, and 0.79 mm in Germany^10^. Additional studies found that mean CIMT was higher in black subjects compared with white subjects aged 35–85 years (0.80 mm vs. 0.75 mm; P < 0.001)^19^ and South Asian subjects aged >45 years (0.64 mm vs. 0.61 mm; P = 0.022)^20^. In line with these studies, we found an elevated mean CIMT with increased age. The age-specific mean CIMT was 0.54 mm in those aged 45–54 years, 0.57 mm in those aged 55–64 years, 0.59 mm in those aged 65–74 years, and 0.61 mm in those aged ≥75 years.

A number of studies have confirmed the association between CIMT and the incidence of stroke in Western and Asian populations^9^,^10^,^11^,^12^,^21^,^22^,^23^,^24^,^25^. Hazard ratios increased from the lowest to the highest tertile of CIMT for every CVD outcome and for all-cause mortality after adjusting for conventional risk factors, carotid plaque groups, and C-reactive protein levels; the highest tertile was associated with an 84% increased risk of composite CVD events, a 54% increased risk of all-cause mortality, and a doubling of risk for CVD-related death^9^. The Rotterdam Study found that for a mean CIMT increase of 0.163 mm, the odds ratios (adjusted for age and sex) were 1.41 (1.25 to 1.82) for stroke and 1.43 (1.16 to 1.78) for myocardial infarction^21^. The Cardiovascular Health Study showed that a maximal CIMT increase of 0.2 mm resulted in age- and sex-adjusted HRs of 1.33 (1.21 to 1.48) for myocardial infarction and 1.37 (1.25 to 1.51) for stroke^22^.

Inconsistent with previous studies, the mean CIMT was lower in this population with a high incidence of stroke, with the quartile of 0.51 mm, 0.55 mm, and 0.61 mm, respectively. The lower level of mean CIMT was in contrast with the higher incidence of stroke and prevalence rates of risk factors, such as hypertension, diabetes, and obesity, in this population^4^,^5^,^6^,^7^,^8^. The prevalence of hypertension was higher in this population than in Taiwanese; with the prevalence of 57.2% for men and 56.2% for women in those aged 45–54 years in this study^7^, while 28.2% of men and 23.6% of women in previous study in Taiwan^18^. However, the mean CIMT in the same age group was 0.54 mm overall, 0.66 mm in men, and 0.61 mm in women in Taiwan. The disparity of low mean CIMT and high incidence of stroke indicates that it is necessary to further evaluate the mean CIMT values in different ethnic populations and regions.

Low socio-economic status, defined as low education, low income, or employed in a manual occupation, has been shown to be associated with increased CIMT^26^,^27^,^28^,^29^,^30^,^31^. Consistent with these findings, mean CIMT decreased by 1.07 × 10^−3^ mm per 1-year increase in education level in this study.

The association between conventional risk factors and CIMT has been established in previous studies; hypertension, diabetes mellitus, smoking, and higher levels of SBP, FBG, TG, and LDL-C have been shown to be associated with increased CIMT^18^,^19^,^20^,^32^,^33^, though an the inverse relationship was found between CIMT and HDL-C^33^,^34^. Similar relationships were found in this study between CIMT and hypertension, current smoking, and higher levels of SBP, FBG, and LDL-C. However, in contrast with previous studies that found a significant correlation between SBP and mean CIMT^35^,^36^,^37^,^38^, a negative correlation was observed between CIMT and DBP and TG in the current study. The exact causes are unknown, and further prospective large population-based studies are needed.

There were several limitations in this study. First, the study population was small and from a local town in Tianjin, China, so the findings may not be generalized to the Chinese population. Second, the cross-sectional study design may have led to a selection bias. However, including only stroke- and CVD-free participants may have reduced this bias. Finally, information regarding medication use and additional blood measurements, such as HDL-C compositions, was absent and may have affected the results. However, lower rates of medication use for hypertension (1.5%) and dyslipidaemia (1.02%) in this population may reduce the impact of medication use on the mean CIMT.

## Conclusions

This study was the first to report the association between CIMT and relevant risk factors in a low-income population with a high incidence of stroke in China. In this study, we evaluated mean CIMT and relevant risk factors amongparticipants aged 45 years and over. There was a disparity between lower mean CIMT and higher incidence of stroke in this population. Established risk factors, including age, sex, education level, hypertension, smoking, SBP, DBP, FBG, and LDL-C, were significantly associated with mean CIMT; however, we observed a negative correlation between mean CIMT and TG. These findings suggest that there are distinct differences in mean CIMT and relevant risk factors between Chinese and Western populations, especially among those with a low socio-economic status. Therefore, it is crucial to explore the primary determinants of stroke and CVD in low-income populations in China in order to decelerate the dramatic increase in stroke incidence and to reduce the burden of stroke.

## Methods

### Study population

This was a population-based cross-sectional study conducted from April 2014 to January 2015. The study population was from the Tianjin Brain Study^5^,^6^,^7^, and the study design was described in a previous study^16^. In brief, the total population included 14,251 persons distributed within 18 administrative villages. Approximately 95% of participants were low-income farmers, with a per capita disposable income of <$1600 US in 2014^39^. All residents aged 45 years and over without CVD were recruited to this study, while those with a previous history of CVD were excluded.

All investigative protocols were approved by the ethics committee of Tianjin Medical University General Hospital and the ethics committee of Peking University First Hospital; the methods were carried out in accordance with the approved guidelines, and informed consent was obtained from all participants.

### Information collection and risk factor definitions

All variables in this study were evaluated by trained epidemiological researchers through face-to-face interviews. A prespecified questionnaire was used to collect all information in this study.

Demographic information, including name, sex, date of birth, and educational level, were obtained from previous records. All participants were categorised into four age groups: 45–54 years, 55–64 years, 65–74 years, and ≥75 years. Educational level was categorised into three groups according to educational years: illiteracy (without education), 1–6 years group, and >6 years group.

Previous individual and family medical histories, which included hypertension, diabetes mellitus, stroke, transient ischemic attack, and coronary heart disease, were obtained according to patient self-reporting or previous records.

Lifestyle characteristics included cigarette smoking, passive smoking, and alcohol consumption. Cigarette smoking was defined as smoking more than 1 cigarette per day for at least 1 year, and participants were categorised as never smokers, ever smokers (ceased smoking for at least 6 months), and current smokers. Alcohol consumption was defined as drinking more than 500 grams of alcohol per week for at least 1 year, and participants were categorised into the never alcohol consumption, ever alcohol consumption (temperance for at least 6 months), and current alcohol consumption groups.

### Physical examination

Measurements of blood pressure (including systolic blood pressure [SBP] and diastolic blood pressure [DBP]), height, and weight were performed in the local village clinic during the baseline survey; levels of fasting blood glucose (FBG), total cholesterol (TC), triglycerides (TG), high-density lipoprotein cholesterol (HDL-C), and low-density lipoprotein cholesterol (LDL-C) in serum were measured at the Ji County People’s Hospital. Carotid ultrasonography and 12-lead echocardiography were also performed by a professional. Body mass index was calculated as weight (kg) divided by the square of height (m^2^).

Hypertension was defined as SBP ≥ 140 mmHg or DBP ≥ 90 mmHg or taking medications for hypertension. Diabetes was defined as FBG ≥ 7.0 mmol/L or taking insulin or oral hypoglycaemic medications.

### Ultrasonography measurements

One trained technician blinded to individuals’ previous disease histories performed all the ultrasound exams. The patients were examined in the supine position using B mode ultrasonography (Terason 3000; Burlington, MA, US) with a 5–12 MHz linear array transducer. CIMTs at the near and far walls of the common carotid artery were measured on the left and right, and 3 values were obtained: the maximum CIMT, minimum CIMT, and average CIMT. Images were obtained and digitally stored according to a standard protocol.

### Statistical analyses

Continuous variables are presented as means with standard deviations, and were compared between groups using the Student’s *t*-test for two groups and analysis of variance for more than two groups. Categorical variables are presented as numbers with frequencies, and were compared using chi-square tests. Multiple linear regression analyses were used to evaluate the associations between traditional risk factors and CIMT. We performed linear regression analyses to evaluate the determinants of CIMT after adjusting for age, educational levels, SBP, DBP, FBG, TC, TG, HDL-C, and LDL-C (continuous variables), and sex, smoking status, alcohol consumption, hypertension, and diabetes (categorical variables). The relationships are presented as unstandardised regression coefficients (β_1_) with standard errors and standardized regression coefficients (β_2_). P values < 0.05 in two-tailed tests were considered statistically significant. SPSS for Windows (version 19.0; SPSS Inc., Chicago, IL, USA) was used for analyses.

## Additional Information

**How to cite this article**: Liu, B. *et al*. Carotid Intima-media Thickness and its Association with Conventional Risk Factors in Low-income Adults: A Population-based Cross-Sectional Study in China. *Sci. Rep.* **7**, 41500; doi: 10.1038/srep41500 (2017).

**Publisher's note:** Springer Nature remains neutral with regard to jurisdictional claims in published maps and institutional affiliations.

## Figures and Tables

**Table 1:** Characteristics of all participants in this study.

Risk factors	Total	Men	Women	P
Total:	3789 (100)	1560 (41.2)	2229 (58.8)	
Age, means (SD), years	59.92 (9.70)	61.13 (9.90)	59.07 (9.47)	<0.001
Age group, n (%)				<0.001
45~54 years	1236 (32.6)	430 (27.6)	806 (35.2)	
55~64 years	1514 (40.0)	632 (40.5)	882 (39.6)	
65~74 years	724 (19.1)	338 (21.7)	386 (17.3)	
≥75 years	315 (8.3)	160 (10.3)	155 (7.0)	
Education, means (SD), years	5.48 (6.54)	6.40 (3.22)	4.84 (3.61)	<0.001
Education, n (%)				<0.001
0 years	659 (17.4)	137 (8.8)	522 (23.4)	
1~6 years	1694 (44.7)	699 (44.8)	995 (44.6)	
>6 years	1436 (37.9)	724 (46.4)	712 (31.9)	
Smoking status, n (%)				<0.001
Never smoking	2840 (75.0)	664 (42.6)	2176 (97.6)	
Ever smoking	173 (4.6)	166 (10.6)	7 (0.3)	
Current smoking	776 (20.4)	730 (46.8)	46 (2.1)	
Alcohol consumption, n (%)				<0.001
Never drinking	3198 (84.4)	999 (64.0)	2199 (98.7)	
Ever drinking	49 (1.3)	48 (3.1)	1 (0.0)	
Current drinking	542 (14.3)	513 (32.9)	29 (1.3)	
Hypertension, n (%)	2583 (68.2)	1111 (71.2)	1472 (66.0)	0.001
Diabetes, n (%)	533 (14.1)	216 (14.1)	317 (14.5)	0.719
Obesity, n (%)	888 (23.4)	323 (20.7)	565 (25.3)	0.001
SBP, means (SD), mmHg	146.42 (22.17)	147.76 (21.41)	145.49 (22.64)	0.002
DBP, means (SD), mmHg	86.81 (11.40)	88.50 (11.22)	85.62 (11.39)	<0.001
BMI, means (SD), Kg/m^2^	25.57 (3.68)	25.20 (3.44)	25.82 (3.82)	<0.001
FBG, means (SD), mmol/L	5.92 (1.57)	5.91 (1.42)	5.93 (1.67)	0.660
TC, means (SD), mmol/L	4.87 (1.09)	4.62 (1.00)	5.04 (1.11)	<0.001
TG, means (SD), mmol/L	1.76 (1.24)	1.61 (1.24)	1.87 (1.22)	<0.001
HDL-C, means (SD), mmol/L	1.46 (0.46)	1.39 (0.43)	1.50 (0.48)	<0.001
LDL-C, means (SD), mmol/L	2.70 (1.25)	2.61 (1.20)	2.76 (1.28)	<0.001

SD, standard deviation; SBP, systolic blood pressure; DBP, diastolic blood pressure; BMI, body mass index; FBG, fasting blood glucose; TC, total cholesterol; TG, triglycerides; HDL-C, high density lipoprotein cholesterol; LDL-C, low density lipoprotein cholesterol.

**Table 2:** Distribution of CIMT by demographical characteristics and risk factors groups.

Risk factors	Numbers	Means (SD)	Statistical magnitude	P
Gender:	3789	0.57 (0.09)	t = 9.487	<0.001
Men	1560	0.58 (0.09)		
Women	2229	0.56 (0.08)		
Age group:			F = 103.534	<0.001
45~54 years	1236	0.54 (0.08)		
55~64 years	1514	0.57 (0.09)		
65~74 years	724	0.59 (0.09)		
≥75 years	315	0.61 (0.09)		
Education:			F = 36.396	<0.001
0 years	659	0.58 (0.09)		
1~6 years	1694	0.57 (0.09)		
>6 years	1436	0.55 (0.08)		
Smoking status:			F = 20.830	<0.001
Never smoking	2840	0.56 (0.09)		
Ever smoking	173	0.58 (0.09)		
Current smoking	776	0.58 (0.10)		
Alcohol consumption:			F = 15.965	<0.001
Never drinking	3198	0.56 (0.09)		
Ever drinking	49	0.60 (0.09)		
Current drinking	542	0.58 (0.10)		
Hypertension:			t = 13.055	<0.001
Yes	2583	0.58 (0.09)		
No	1206	0.54 (0.08)		
Diabetes:			t = 3.812	<0.001
Yes	533	0.58 (0.09)		
No	3192	0.56 (0.09)		
BMI:			t = 1.147	0.318
Normal	1298	0.57 (0.09)		
Overweight	1603	0.57 (0.09)		
Obesity	888	0.57 (0.08)		
TC ≥ 6.22 mmol/L:			t = 0.242	0.808
Yes	389	0.57 (0.08)		
No	3336	0.57 (0.09)		
TG ≥ 2.26 mmol/L:			t = 2.292	0.022
Yes	816	0.56 (0.08)		
No	2909	0.57 (0.09)		
HDL-C ≤ 1.04 mmol/L:			t = 1.441	0.150
Yes	546	0.57 (0.09)		
No	3179	0.57 (0.09)		
LDL-C ≥ 4.14 mmol/L:			t = 0.236	0.019
Yes	296	0.58 (0.10)		
No	3429	0.57 (0.09)		

TC, total cholesterol; TG, triglycerides; HDL-C, high density lipoprotein cholesterol; LDL-C, low density lipoprotein cholesterol.

**Table 3:** Multivariate analysis for the risk factors of earlier AS in this study (×10^−3^ mm).

Reference group	Regression coefficients (95% CI)	Standardized regression coefficients	P
Age	1.66 (1.30, 2.01)	0.182	<0.001
Gender (Male vs. Female)	19.03 (11.61, 26.44)	0.106	<0.001
Education	−1.07 (−1.94, −1892.82)	−0.043	0.017
Smoking:			
Ever smoking vs. Never smoking	−6.98 (−21.02, 7.06)	−0.017	0.330
Current smoking vs. Never smoking	9.82 (1.14, 18.51)	0.045	0.027
Drinking alcohol:			
Ever drinking vs. Never drinking	10.52 (−14.23, 35.28)	0.013	0.405
Current drinking vs. Never drinking	6.70 (−2.27, 15.67)	0.027	0.143
Hypertension vs. No hypertension	18.07 (10.23, 25.92)	0.095	<0.001
SBP	0.40 (0.21, 0.60)	0.101	<0.001
DBP	−0.37 (−0.71, −0.03)	−0.048	0.032
FBG	2.24 (0.50, 3.98)	0.040	0.001
TG	−2.36 (−4.56, −0.16)	−0.033	0.035
LDL-C	4.95 (2.81, 7.09)	0.070	0.003

95% CI: 95% confidence interval.
